# Review of patients with achondroplasia: a single-center's experience with follow-up and associated morbidities

**DOI:** 10.1007/s00431-024-05643-y

**Published:** 2024-06-15

**Authors:** Merve Soğukpınar, Gizem Ürel Demir, Gülen Eda Utine, Elmas Nazlı Gönç, Zeynep Alev Özön, Pelin Özlem Şimşek-Kiper

**Affiliations:** 1 Division of Pediatric Genetics, Department of Pediatrics, Faculty of Medicine, Hacettepe University, Ankara, Turkey; 2 Division of Pediatric Endocrinology, Department of Pediatrics, Faculty of Medicine, Hacettepe University, Ankara, Turkey

**Keywords:** Achondroplasia, *FGFR3* heterozygous variant, Morbidity, Mortality

## Abstract

Achondroplasia (ACH; MIM #100,800), caused by a heterozygous gain of function pathogenic variant in the fibroblast growth factor receptor 3 gene (*FGFR3*; MIM*134,934), is the most prevalent and most readily identifiable cause of disproportionate short stature that is compatible with life. In addition, individuals with achondroplasia face significant medical, functional, and psychosocial challenges throughout their lives. This study assessed associated morbidities in patients with achondroplasia at a single center in Turkey. In this study, the clinical findings and associated morbidities of a group of patients with achondroplasia (n = 68) with clinical multidisciplinary follow-up at a single center between the years 2005–2023 are evaluated retrospectively. A total of 68 patients, 30 male (44.1%) and 38 female (55.9%), were evaluated. In the majority (84.2%) of patients, shortness of extremities was detected in the prenatal period at an average of 28.7 gestational weeks (± 3.6 SDS) with the aid of ultrasonography. More than half (n = 34/63, 54%) of the patients had a father of advanced paternal age (≥ 35 years). Among the complications, respiratory system manifestations, including obstructive sleep apnea (70%), ear-nose-throat manifestations including adenoid hypertrophy (56.6%) and otitis media (54.7%), neurological manifestations due to foramen magnum stenosis (53.2%), and skeletal manifestations including scoliosis (28.8%), are represented among the most common. The mortality rate was 7.3% (n = 5/68).

*Conclusion*: This study not only represents the first retrospective analysis of the associated morbidities of patients with achondroplasia from a single center in Turkey but also will provide a reference point for future studies.

## Introduction

**Fig. 1:**
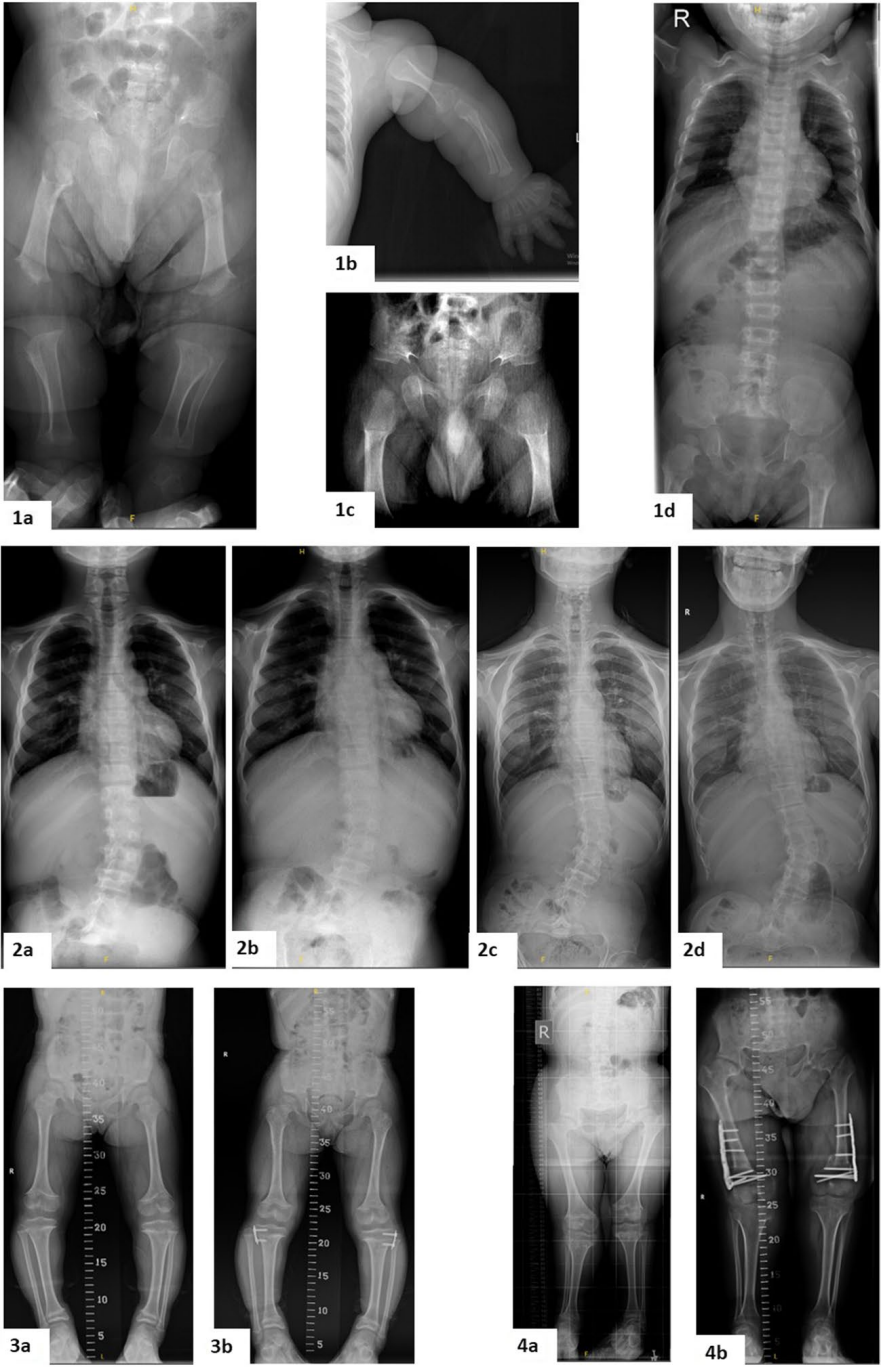
The typical radiographic findings in ACH. Please note; 1a) generalized metaphyseal changes and proximal femoral radiolucency; 1b) short tubular bones, rhizomelic shortening of the arms, and trident hand; 1c) proximal femoral radiolucency and narrow sacrosciatic notch; 1d) narrowing of the interpedincular distance of the caudal spine in infancy period; 2a-2d) progressive scoliosis in a patient aged 6, 9, 16, and 19 years; 3a-3b) genu varum in a patient and postoperative radiograph aged 8–9 years; 4a-4b) genu valgum in a patient and postoperative radiograph aged 15–17 years

**Fig. 2:**
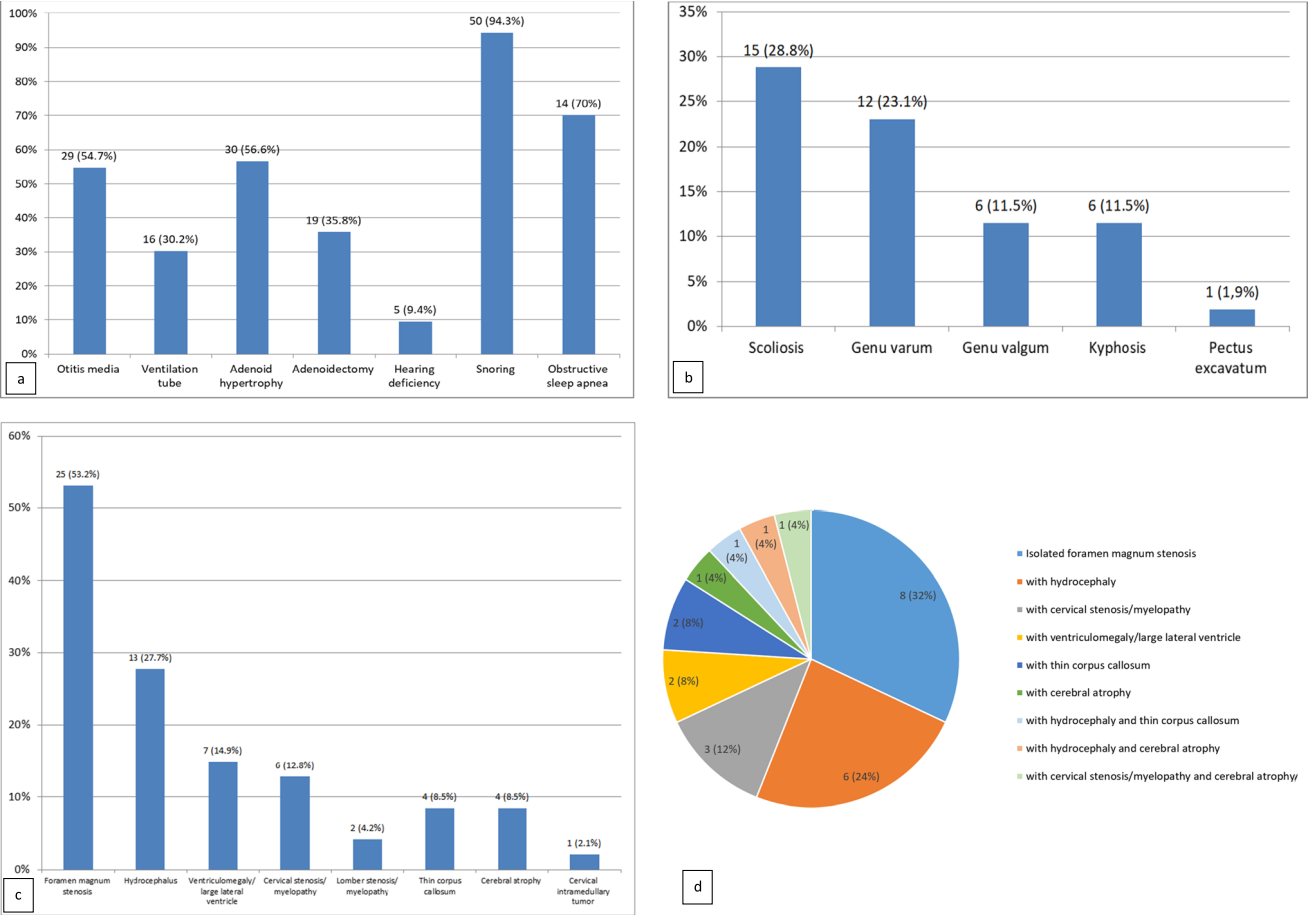
Complications observed in patients with ACH are presented. a. ENT and respiratory manifestations (n = 53), b. Skeletal manifestations (n = 52), c. Neuroimaging findings (n = 47), d. The most common finding foramen magnum stenosis and other associated central nervous system abnormalities (n = 25)

Achondroplasia (ACH; MIM#100,800) is the most common cause of disproportionate short stature worldwide. According to the best estimates, it occurs in 1:25,000–1:30,000 live births [1]. The most characteristic clinical findings include rhizomelic shortening of the limbs, macrocephaly, and characteristic facial features with a low nasal bridge, frontal bossing, and midface hypoplasia. Hypotonia is typical in infancy and early childhood, and the acquisition of motor developmental milestones is often delayed [2]. Nevertheless, intelligence is generally normal. ACH is caused by heterozygous pathogenic variants in the fibroblast growth factor receptor 3 gene (*FGFR3*, MIM*134934), a transmembrane receptor tyrosine kinase [3, 4]. About 98% of patients with ACH have the variant c.1138G > A, while only 1% have c.1138G > C, both of which cause a glycine to arginine substitution at amino acid position 380, p.Gly380Arg. In 80% of cases, ACH is sporadic, and the disease is due to a de novo pathogenic variant [5]. Such de novo variants occur exclusively in the father’s germline and increase in frequency with advanced paternal age (> 35 years) [6]. The diagnosis of ACH can be established on the basis of clinical and radiographic features (short tubular bones, generalized metaphyseal changes, proximal femoral radiolucency, narrowing of the interpedincular distance of the caudal spine, and narrow sacrosciatic notch) (Fig. 1). ACH requires multidisciplinary follow-up in order to prevent life-threatening complications and increase the quality of life [7–9]. Complications such as craniocervical junction compression, central and obstructive sleep apnea, recurrent otitis media, conductive hearing loss, bowing of the lower legs, kyphosis, spinal stenosis, and obesity may occur [7, 10] (Fig. 2). The overall life expectancy is decreased by approximately 10 years in the present adult ACH population [11, 12]. In this study, we aimed to evaluate the associated morbidities of patients with ACH who are under follow-up at a single center in Turkey.

## Materials and methods

Patients with ACH, regardless of molecular confirmation, who were under clinical follow-up at Hacettepe University Faculty of Medicine, Department of Pediatric Genetics between the years 2005–2023, were included in the study. The prevalence of developmental and behavioral, neurologic, respiratory, ear and hearing, musculoskeletal, and dental findings were recorded and analyzed. Neuroimaging findings were evaluated when magnetic resonance imaging (MRI) scans were available. Data were gathered retrospectively from hospital records. Information regarding the age at diagnosis, parental ages, body mass index, developmental assessment (formal/verbal), molecular tests, and history of recurrent otitis media and/or ventilation tube insertion, adenoid hypertrophy and/or adenoidectomy, hearing loss, snoring, obstructive sleep apnea, cardiac anomalies, skeletal and neurological symptoms, surgeries (neurosurgery/orthopedic), and mortality were recorded. Associated morbidities detected in patients were divided into two age groups, childhood and adult (> 18 years), according to the current age of the patient at the time of this study.

The Denver II developmental screening test had been used for the standard assessment of development, in patients with ACH younger than 6 years at our institution. Patients older than 6 years of age were assessed cognitively by the use of the WISC-R (Wechsler Intelligence Scale for Children-Revised).

The diagnosis of hydrocephalus was established through the evaluation of various radiographic parameters, including ventricular dilation (Evans' index > 0.3), narrow sulci, distension of the third ventricular recesses, bulging third ventricular floor, elevated corpus callosum, reduced mamillopontine distance, widening of the lateral ventricular horns, narrowing of the ventricular angle, and aqueductal flow void on MRI examinations [13]. Scoliosis examination was performed by specifically measuring the Cobb angle on X-rays to determine the degree of spinal curvature. A diagnosis of scoliosis was made when a coronal curvature assessed on a posterior-anterior radiograph exceeded 10 degrees. Foramen magnum stenosis was evaluated based on the presence of MRI findings, including narrowing of the craniocervical junction, loss of cerebrospinal fluid space, cord compression, and increased T2 cord signal [14].

Body mass index (BMI) was assessed according to the Centers for Disease Control and Prevention (CDC) guidelines for those aged 2 to 20 years [15]. When compared to the normal population, 5–85 percentile were considered healthy weight, 85–95 percentile overweight, and ≥ 95 percentile obese in the 2–20 age group. For patients over 20 years, a BMI between 25 and 30 was defined as overweight, and a BMI of 30 or higher as obese. Patients classified as overweight or obese according to normal population data were then re-evaluated according to the BMI charts for patients aged 0–16 years with ACH, as detailed in the study by Hoover-Fong et al. [16].

Statistical analyses were performed using SPSS for Windows Version 22.0. Numerical variables were summarized by mean ± standard deviation, discrete numeric variables were expressed as medians (minimum–maximum), and qualitative variables were summarized by number and percentage.

## Results

The data of 68 patients with ACH were analyzed retrospectively. Clinical findings, demographic features, and associated morbidities of the patients are presented in Table 1 and Fig. 1–2.

**Table 1:** The demographic and clinical features of all patients with ACH

Patients with ACH (n = 68)		Affected / Data available patient (n,%)
Demographic features	Age at diagnosis, mo (median, min–max)	2 (0–153)
	Gender (female/male)	38/30
	Advanced paternal age (≥ 35 years)	34/63 (54%)
	Consanguinity	15/68 (22.1%)
	Parental history of ACH	4/68 (5.9%)
Shortening of extremities on prenatal ultrasonography		48/57 (84.2%)
Molecular diagnosis	*FGFR3*	
	c.1138G > A; p.Gly380Arg	30/31 (96.8%)
	c.1031C > G; p.Ser344Cys	1/31 (3.2%)
ENT manifestations and interventions (n = 53)	Otitis media	29/53 (54.7%)
	Ventilation tube	16/53 (30.2%)
	Adenoid hypertrophy	30/53 (56.6%)
	Adenoidectomy	19/53 (35.8%)
	Hearing deficiency	5/53 (9.4%)
Respiratory manifestations (n = 53)	Snoring	50/53 (94.3%)
	Apnea (informal assesment)	16/53 (30.2%)
	Obstructive sleep apnea (with PSG)	14/20 (70%)
Skeletal manifestations (n = 52)	Scoliosis	15/52 (28.8%)
	Genu varum	12/52 (23.1%)
	Kyphosis	6/52 (11.5%)
	Genu valgum	6/52 (11.5%)
	Pectus excavatum	1/52 (1.9%)
Orthopedic surgery (n = 52)	Lengthening surgery	6/52 (11.5%)
	Fixation osteotomy	3/52 (5.8%)
Neurological manifestations and neuroimaging findings (n = 47)	Headache	6/47 (12.8%)
	Arm neurological signs (pain/ paresthesia /weakness)	0/47 (0%)
	Leg neurological signs (pain/ paresthesia /weakness)	2/47 (4.2%)
	Foramen magnum stenosis	25/47 (53.2%)
	Hydrocephalus	13/47 (27.7%)
	Ventriculomegaly/ large lateral ventricle	7/47 (14.9%)
	Cervical stenosis / myelopathy	6/47 (12.8%)
	Lomber stenosis / myelopathy	2/47 (4.2%)
	Thin corpus callosum	4/47 (8.5%)
	Cerebral atrophy	4/47 (8.5%)
	Cervical intramedullary tumor	1/47 (2.1%)
Neurosurgical interventions (n = 47)	Cervical laminectomy	2/47 (4.2%)
	Cervical decompression	2/47 (4.2%)
	Thoracolumbar laminectomy	1/47 (2.1%)
	Shunt surgery	1/47 (2.1%)
	Total	6/47 (12.7%)
Other findings	Nephrolithiasis	1/68 (1.5%)
	Hydronephrosis	1/68 (1.5%)
	Ectopic kidney	1/68 (1.5%)
	Gastroesophageal reflux	4/68 (5.9%)
	Constipation	5/68 (7.3%)
	Swallowing difficulty	3/68 (4.4%)
	Hypogammaglobulinemia	2/68 (2.9%)
	Down syndrome	1/68 (1.5%)
	Craniosynostosis	1/68 (1.5%)
	Acanthosis nigricans	1/68 (1.5%)
	Hypothyroidism	2/68 (2.9%)
	Hypergonadotropic hypogonadism and streak gonad	1/68 (1.5%)
	Syndrome of inappropriate antidiuretic hormone (SIADH) secretion	1/68 (1.5%)
Mortality	5/68 (7.3%)	

mo: months; ENT: Ear-Nose-Throat; PSG: Polysomnography

### Demographic findings

Thirty patients were male (44.1%), and 38 were female (55.9%), with a male-to-female ratio of 0.79. Advanced paternal age (≥ 35 years) was observed in 34 patients (54%). Parental consanguinity was present in 15 patients (22%). Upon examining the last recorded ages of our patients, the youngest was 5 months old, and the oldest was 28 years old.

### Prenatal findings

The finding of shortness of extremities was detected in 84.2% of the patients during the prenatal period, at an average of 28.7 weeks (± 3.6 SD) with the aid of ultrasound. Macrocephaly was found in 10 patients, a narrow thorax in one patient, and polyhydramnios in two patients with shortness of extremities. Premature birth occurred in 10 patients (between 30–35 weeks).

### Diagnostic findings

While the median age at clinical diagnosis was 2 months (range: 0–153 months), the median age at molecular diagnosis was 12 months (range: 0–336 months). One patient had a molecular diagnosis with a prenatal test. Sanger sequencing results for 31 patients (45.6%) were available. Almost all (n = 30/31, 96.8%) had a heterozygous pathogenic variant in *FGFR3* c.1138G > A;p.Gly380Arg. One patient had a maternally inherited heterozygous, and previously reported *FGFR3* c.1031C > G; p.Ser344Cys pathogenic variant [4]. The majority of cases were sporadic. A family history of ACH was positive in only four patients (n = 4/68, 5.9%).

### Ears-hearing and respiratory findings (n = 53)

Twenty-nine patients with ACH (54.7%, n = 29/53) had recurrent otitis media, and a ventilation tube was inserted in 16 (55.2%, n = 16/29). Thirty patients exhibited adenoid hypertrophy and 19 of them underwent adenoidectomy (63.3%, n = 19/30). Five patients experienced hearing deficiency (9.4%, n = 5/53). There were no patients presenting with ear, nose, and throat findings for the first time in adulthood. In terms of symptoms, 50 patients (94.3%) reported snoring, and 16 experienced apnea (30.2%). Obstructive sleep apnea was diagnosed in 14 of the 20 patients (70%) who underwent polysomnography (PSG) as part of routine follow-up. The families of two of these patients did not report any instances of apnea. Nine patients had been using CPAP (continuous positive airway pressure) therapy. Three patients persisted in snoring throughout adulthood; however, they did not describe apnea clinically, and PSG was not performed in adulthood.

### Growth and developmental findings

**Fig. 3:**
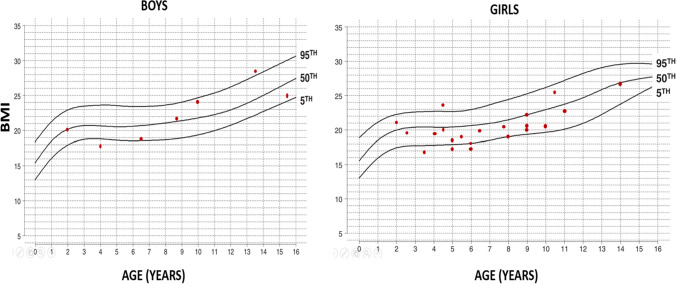
BMI curves (5th, 50th, and 95th percentiles) of 28 patients from 0 to 16 years of age in boys and girls with achondroplasia considered overweight or obese compared to normal population (The BMI values of the patients with ACH in the last control are shown as a red dot on the curve) [16]

Anthropometric measurements of the patients were evaluated according to ACH-specific growth curves [17]. Among our patients, the maximum height was 135 cm for men and 123 cm for women. Of the patients who were over 2 years at the last check-up and for whom height and weight data were available (n = 37/68, 54.4%), thirteen were overweight and eighteen were obese. However, when 28 of these 31 patients aged 0–16 years were evaluated according to the ACH BMI curve, only 2 patients (5.4%) had a BMI above the 95th percentile **(**Fig. 3**)**. The other three patients were 19, 19, and 27 years old at last visit with BMIs of 32.4, 27.4, and 30.6, respectively. Of the 68 patients, 20 underwent a formal assessment of development (n = 20/68, 29.4%). When assessing the developmental status of patients with ACH, a specific chart was not used, but developmental delay based on physical differences was taken into account in patients with ACH compared to similar age matched groups. Denver II developmental screening test results were available for 18 patients. Among these 18 patients, seven (38.9%) exhibited developmental delays in at least two of the motor, language and social domains, five (27.8%) had gross motor delay, two (11.1%) experienced language delay, one (5.6%) showed social delay, and three (16.7%) had normal development. During the follow-up period, the WISC-R was administered to eight of these patients. In total, the WISC-R was performed on 10 patients (14.7%, n = 10/68), specifically in those with a suspicion of learning or intellectual disability after reaching school age. Of these 10 patients, two had normal results, four were found to have borderline intelligence, one had mild intellectual disability, one had moderate intellectual disability, and two had learning disabilities. It is also important to mention that the WISC-R may underestimate performance at patients with ACH because it is based on motor skills.

In the informal assessment of developmental stages, which involved 49 of the 68 patients, 36.7% were found to have developmental delays in at least two areas, primarily motor and language; 36.7% had isolated motor delay, 4.1% had isolated language delay, and 22.4% showed normal development.

### Neurological and neurosurgical findings (n = 47)

Central nervous system imaging revealed foramen magnum stenosis in 25 patients (53.2%), hydrocephalus in 13 patients (27.7%), ventriculomegaly/large lateral ventricles in seven patients (14.9%), cervical stenosis and/or myelopathy in six patients (12.8%), lomber stenosis and/or myelopathy in two patients (4.2%), a thin corpus callosum in four patients (8.5%), cerebral atrophy in four patients (8.5%), and a cervical intramedullary tumor in one patient (2.1%). Five patients (7.3%, n = 5/68) underwent surgical intervention; two underwent cervical laminectomy (one had severe foramen magnum stenosis, and the other had a cervical intramedullary mass), two had cervical decompression surgery (one had severe foramen magnum stenosis and apnea, and the other had a narrow craniocervical junction and hydrocephalus), and one patient had a thoracolumbar laminectomy due to spinal canal stenosis at the T11-L1 level. Among these patients, only one required VP shunt intervention. In adulthood, only one patient who presented with leg pain and paresthesia had lumbar stenosis and myelopathy and did not require surgery.

### Musculoskeletal findings (n = 52)

Kyphosis persisting after infancy was observed in six patients (11.5%), genu varum in 12 (23.1%), genu valgum in six (11.5%), scoliosis in 15 (28.8%), and pectus excavatum/carinatum in one patient (1.9%). Fixation osteotomy was performed on one patient for genu valgum, and on two patients for genu varum. Lengthening surgery was performed on six of the 52 patients (11.5%). Some of the radiographic findings are shown in Fig. 1. Upon evaluation in adulthood, one patient had genu varum and one patient had scoliosis. We did not have any patients with a history of orthopedic surgery during this period.

### Occasional findings

Other findings observed in our patients, which were not anticipated during the routine follow-up for ACH, included gastroesophageal reflux, constipation, swallowing difficulty, nephrolithiasis, hydronephrosis, ectopic kidney, hypothyroidism, syndrome of inappropriate antidiuretic hormone (SIADH) secretion, hypergonadotropic hypogonadism and streak gonad, hypogammaglobulinemia, craniosynostosis, and acanthosis nigricans **(**Table 1**).**

### Mortality

In our study, five of the 68 patients died, yielding a mortality rate of 7.3%. These patients died at 5 months, 12 months, 16 months, 24 months, and 36 months, all with a diagnosis of pneumonia. One individual had both ACH and Down syndrome, experienced obstructive apnea, pulmonary hypertension and other sequelae. The lethal episode arose secondary to aspiration. Another patient, died at 12 months of age; surgery for a cervical intramedullary mass was followed by postoperative pneumonia. The other three patients also had histories of recurrent pneumonia; additionally, one of them had severe sleep apnea, and another had both pulmonary hypertension and difficulty swallowing. It is not clear why in our population life-threatening pneumonia appears to be so much more frequent than is typically reported in ACH. We did not have any patients who were followed into advanced ages and died in adulthood.

## Discussion

ACH is the leading cause of disproportionate short stature, and individuals with ACH encounter various medical, functional, and psychosocial challenges over their lifetimes. Early intervention and regular follow-up are essential for effectively managing potential complications in individuals with ACH. This study aimed to identify the most common clinical features and associated morbidities among 68 patients with ACH diagnosed in our clinic over the past 25 years.

Short stature is one of the main concerns in ACH. Moderate to marked short stature is present in all affected individuals. Obesity is another concern in ACH [18]. However, excessive weight gain usually becomes noticeable during early childhood, and in adulthood, obesity can exacerbate morbidity associated with lumbar stenosis, cause generalized joint issues, and contribute to the onset of cardiovascular complications [12]. Childhood obesity rates in ACH have been estimated to range from 0 to 10%, which is significantly lower than the rates observed in adults. Among adolescents, the prevalence of overweight and obesity has been reported to be as high as 56% [19, 20]. In our study, we evaluated the body mass index of 37 patients with ACH during the most recent follow-up. In the age group of 0–16 years, only two patients were obese according to the standards of ACH. However, three patients for whom BMI data were available in adulthood, one was overweight and two were obese. Although this suggests that the prevalence of adult obesity is increasing, the number of adult patients was insufficient for a clear interpretation.

Individuals with ACH generally have normal cognitive development and function [21]. However, when compared with controls, they have specific differences in development, including delayed and unusual motor development and language-related problems [22]. Mild to moderate hypotonia is typical during infancy, making it difficult for infants to support their heads. This, along with variations in body habitus, contributes to motor development delays [23–25]. Except in cases of hydrocephalus or other central nervous system problems, intelligence is normal [26]. Nevertheless, a small minority of children with ACH will be more seriously delayed, demonstrate significant learning disabilities, and may have autism spectrum disorders and/ or a cognitive disability [22]. Although the frequency of such problems has not yet been well documented, it is suggested that it accounts no greater than 10% [22]. In this study, the majority of patients initially had a delay in gross motor skills during infancy, as noted in informal assessments. However, they eventually caught up with their peers. Formal evaluations, using either the Denver II Developmental Screening Test or WISC-R, indicated that four patients had borderline intelligence, one had mild intellectual disability, one had moderate intellectual disability, and two (a twin pair) had learning disabilities and attention deficit hyperactivity disorder (ADHD). Notably, two additional patients (n = 2/68, 2.9%) were also found to have serious delays. The brain MRIs of these patients showed foramen magnum stenosis with hydrocephalus in four patients, cervical stenosis in two patients, cervical stenosis and cerebral atrophy in one patient, and thin corpus collosum in one patient.

Symptomatic hydrocephalus is rare in ACH [7, 27, 28]. However, around 5% of children with ACH may develop symptomatic increased intracranial pressure requiring intervention [29]. The reported percentage of individuals requiring treatment with a VP shunt or a subdural tap ranged between 2–5% [30–32]. In the present study, about one third (n = 13/47, 27.7%) of patients had hydrocephalus. However, VP shunt surgery was required in only one (7.7%) patient.

Population-based studies indicate that without evaluation and treatment, the excess risk of death for infants with ACH could reach 7.5% in the first year of life due to issues with the craniocervical junction [11]. This risk seems to be related to central apnea caused by damage to the respiratory control centers. This risk could be reduced to as little as 0.3% with evaluation and neurosurgical treatment [33]. In the present study, following a neurological evaluation and cranial MRI, a total of five patients (n = 5/47, 10.6%) with ACH underwent intervention.

Foramen magnum stenosis is a well-recognized, serious, and potentially life-threatening complication in ACH. It might clinically present with sleep-disordered breathing, hypotonia, or hypertonia with increased reflexes and extensor plantar responses; however, children and infants with foramen magnum stenosis can also be asymptomatic [7]. To ensure optimal monitoring of this potentially life-threatening complication, guiding principles have been developed [34]. These include routine clinical monitoring of infants and young children, scheduled magnetic resonance imaging screenings, referral of suspected cases to a neurosurgeon, combined assessments to inform decompression decisions, collaborative decision-making on proceeding with decompression, and management strategies for older children with previously undetected foramen magnum stenosis [34]. In the present study, foramen magnum stenosis was detected in more than half (53.2%) of the patients, yet six of them (24%) had headache, nine had apnea (36%), and three (12%) required surgical intervention. Risks for apnea-related death, as well as high cervical myelopathy and paralysis, result from the foramen magnum's growth being out of phase with that of the spinal cord. High cervical myelopathy can also be caused by compression of the cervicomedullary cord, typically manifesting in young children as disproportionate and persistent hypotonia, weakness, asymmetric reflexes, and hyperreflexia [35]. Therefore, from the time of diagnosis, every patient should undergo a thorough neurological examination, neuroimaging, and PSG evaluation. Symptomatic spinal stenosis, affecting L1-L4, is the most prevalent medical condition in adults [36]. In our ACH group, headache was noted in six patients; arm pain, weakness, and paresthesia in one; and leg pain, weakness, and paresthesia in two patients. These symptoms frequently manifested in late adolescence and adulthood.

Thoracolumbar junction kyphosis affects 89% of infants with ACH, but it usually resolves spontaneously over time [37]. The prevalence of kyphosis is lower, ranging from 19 to 35%, in children of walking age (over 3 years) and adolescents [38]. In our study, six patients (11.5%) exhibited kyphosis at ages over 3 years. Scoliosis is another common skeletal finding, affecting 60% of patients at an average age of 18 years [39]. In the present study, scoliosis was the most common musculoskeletal finding, observed in 28.8% of patients, yet none of the patients required surgical intervention. Only three patients had been using a corset for scoliosis. Scoliosis in patients was determined by considering both clinical and radiographic findings, however, the lower incidence of scoliosis among our patients was thought to be due to their younger average age (median: 6 years; range: 0–28 years) and missing follow-up data.

Otitis media and hearing loss are common problems in ACH [22]. Otitis media affects about 80% of all children with ACH at some point in their lives. In the present study, more than half (54.7%) of the patients had otitis media, and 9.4% experienced hearing loss. Previous studies reported higher rates of otitis media (80%) and hearing loss (37%) in patients with ACH than in our patients, which was thought to be related to missing data of some patients not continuing their regular follow-up after diagnosis [40]. Of patients with otitis media, about one-third (30.2%) had a history of ventilation tube placement. Snoring, mouth breathing, sleep apnea, and obstructive sleep apnea are common breathing disorders in ACH. Among our patients, snoring was a very common symptom (94.3%). In their clinical histories, apnea accompanying snoring was observed in 16 patients (30.2%). Obstructive sleep apnea (OSA) was detected in 14 of 20 patients for whom PSG evaluation data were available. PSG should be performed on all patients with ACH following diagnosis, since clinical history is a poor predictor of apnea [41]. Of note, despite the absence of symptoms such as snoring or apnea, OSA was detected with PSG in two of our patients.

Approximately half of patients (n = 31; 45.6%) have undergone at a minimum one surgery related to ACH such as ventilation tube insertion, adenoidectomy, neurosurgery, ortopedic surgery. This rate was 80% in the United States, 75.7% in the Japanese, and 72% Europe ACH cohort [42–44]. It has been suggested that inadequate patient data may be the reason for the lower rate of surgery in this study.

Middle ear procedures were the most prevalent type of surgeries as in the previous studies [42–44]. Lengthening surgery was performed in six patients (11.5%) in our cohort. The rate of lengthening surgery in patients with ACH varies considerably among countries, ranging from 1.2% in the United States to ~ 60% in Japan and up to 90% in ~ Spain [42]. Besides medical approaches, societal perspectives on disease and cultural values are likely to play a role in the development of such serious differences. For the same considerations, the rate of growth hormone use in treatment in a study in Japan was 75.7%, whereas in this study, three patients (4.4%) received growth hormone therapy [44].

Interestingly, a few unusual findings in some of our patients caught our attention. In one of the twin pairs, headache, learning difficulties, and ADHD were present and a brain MRI displayed foramen magnum stenosis and craniocervical junction compression. The fundus examination revealed papilledema, but neurological examination was completely normal, and an increase in intracranial pressure was not evident. Nevertheless, after receiving medical treatment with acetazolamide, the papilledema improved. Patients with ACH with papilledema have previously been reported; however, unlike our patients in discussion, hydrocephalus and high intracranial pressure were generally present in these patients [45].

The other interesting finding was a patient with hypergonadotropic hypogonadism and streak gonads with a karyotype of 46,XX. To the best of our knowledge, gonadal dysgenesis has not previously been reported in ACH. Our patient exhibited hypogammaglobulinemia and severe intellectual disability as well. Due to atypical findings, advanced molecular analysis including chromosomal microarray analysis and exome sequencing was performed in addition to *FGFR3* sequence analysis. Apart from the *FGFR3* c.1138G > A heterozygous variant, no other pathogenic or clinically significant variant was identified through these tests.

Lastly, during follow-up, one of the patients, who showed both phenotypic and radiological findings consistent with ACH, displayed acanthosis nigricans and global developmental delay which suggested a diagnosis of severe ACH with developmental delay and acanthosis nigricans (SADDAN; MIM#616482). Physical examination revealed diffuse hyperpigmentation on the neck and trunk with dryness of the skin. There was no evidence of insulin resistance or adrenal insufficiency. Therefore, the most common variant in SADDAN c.1949A > T; p.Lys650Met was initially checked which was normal. A heterozygous *FGFR3* pathogenic variant, c.1138G > A was detected in this patient.

Acanthosis nigricans in patients with ACH has rarely been reported in the literature; however, a recent study showed that it is present in 10% of patients. It is more likely to occur in the non-white population and has typically been observed to first appear in prepubertal or adolescent years [46]. In our patient group, only one patient (1.5%) had acanthosis nigricans, which may be related to the patients' ages and the fact that some of the patients admitted were from the non-white population. At the last examination, 54 patients (79.4%) were preadolescents (under 10 years old), and we plan to continue monitoring these patients for acanthosis nigricans.

In previous studies, mortality in children under 4 years of age was often sudden death with acute brainstem compression [12, 33]. The five patients (7.3%) who deceased in our study were also due to pneumonia. One of them had Down syndrome and died due to an aspiration pneumonia and the other one died due to a postoperative pneumonia. Although recurrent pneumonia and serious infections are an expected finding in Down syndrome, life-threatening pneumonia in patients with ACH is not as common as it was in our study. These patients had symptoms such as severe sleep apnea and difficulty swallowing, so we thought they might have recurrent infections associated with complications secondary to foramen magnum stenosis. However, we did not have data to support this because two of our five patients who died did not have a brain MRI before death and the other three did not have foramen magnum stenosis.

This study has several limitations, one of which is the missing follow-up data for some of our patients. Although this study includes patients of various ages, we were unable to plot the distribution of height SD scores due to missing data. Secondly, formal assessments for clinical findings, including intellectual disability and apnea, were not available for some patients. Thirdly, information on multidisciplinary and adulthood follow-up is missing.

In conclusion, ACH is commonly viewed as a mild condition, yet its associated health risks and potential for mortality are often underestimated. Early and continuous monitoring for serious complications from diagnosis onwards is essential to prevent early death, handle future health issues, and enhance the overall quality of life. Despite being the most prevalent form of non-lethal skeletal dysplasia, ACH's complications are not limited to orthopedic problems alone. This highlights the need for a comprehensive approach by a multidisciplinary healthcare team to cater to the varied needs of individuals with ACH.

## Data Availability

No datasets were generated or analysed during the current study.

## Abbreviations

*ACH*: Achondroplasia
*ADHD*: Attention deficit hyperactivity disorder
*BMI*: Body mass index
*CDC*: Centers for Disease Control and Prevention
*CPAP*: Continuous positive airway pressure
*MRI*: Magnetic resonance imaging
*OSA*: Obstructive sleep apnea
*PSG*: Polysomnography
*SADDAN*: Severe achondroplasia with developmental delay and acanthosis nigricans
*SIADH*: Syndrome of inappropriate antidiuretic hormone
*WISC-R*: Wechsler Intelligence Scale for Children-Revised
*VP shunt*: Ventriculo-peritoneal (VP) shunt
