# Correction to: Review of patients with achondroplasia: a single-center's experience with follow-up and associated morbidities

**DOI:** 10.1007/s00431-024-05688-z

**Published:** 2024-07-18

**Authors:** Merve Soğukpınar, Gizem Ürel Demir, Gülen Eda Utine, Elmas Nazlı Gönç, Zeynep Alev Özön, Pelin Özlem Şimşek-Kiper

**Affiliations:** 1 Division of Pediatric Genetics, Department of Pediatrics, Faculty of Medicine, Hacettepe University, Ankara, Turkey; 2 Division of Pediatric Endocrinology, Department of Pediatrics, Faculty of Medicine, Hacettepe University, Ankara, Turkey

**Correction to: European Journal of Pediatrics**

10.1007/s00431-024-05643-y

During the typesetting of the published article entitled “Review of patients with achondroplasia: a single center's experience with follow up and associated morbidities”, there was an error in the affiliation of author Pelin Özlem Şimşek-Kiper.

Published version:

Pelin Özlem Şimşek Kiper^2^

^2^Division of Pediatric Endocrinology, Department of Pediatrics, Faculty of Medicine, Hacettepe University, Ankara, Turkey

Corrected form:

Pelin Özlem Şimşek Kiper^1^

^1^Division of Pediatric Genetics, Department of Pediatrics, Faculty of Medicine, Hacettepe University, Ankara, Turkey

The original article has been corrected.

